# Abstract of Researches on Magnetism and on Certain Allied Subjects, Including a Supposed New Imponderable

**Published:** 1846-04

**Authors:** 


ABSTRACT OF " RESEARCHES ON MAGNETISM AND ON CERTAIN ALLIED SUBJECTS, INCLUDING A SUPPOSED NEW IMPONDERABLE. By Baron *Von Reichenbach.* Translated from the German by *W. Gregory*, M.D. F.R.S.E., &c. 8vo. pp. 112. London, 1846. Taylor and Walton.

The highly respected names of the author and of the translator (the present Professor of Chemistry in the University of Edinburgh) of this memoir will justify us in briefly noticing it, however extraordinary, nay extravagant, many of the statements which it contains may seem to be. Without presuming to determine what amount of credit is to be attached to them, we shall, for the present, do nothing more than give a very short summary of the principal positions that are attempted to be established.

Magnets of a certain power, when drawn along the body, without contact, produce the sensation of an *aura,* accompanied with a feeling of a pricking or formication of the skin, and sometimes with headache, convulsive spasms of different parts and so forth. Females are much more readily affected in this respect than males; and those of a nervous temperament than those in robust health. Epileptic, cataleptic, and hysterical patients are particularly sensitive; and lunatics and somnambulists are uniformly so. Highly sensitive or impressionable persons are sometimes seized with fainting and violent spasms, on the approach of a magnet: at the same time, the acuteness of their smell, taste, and vision becomes wonderfully exalted. When this is the case, they perceive, in the dark, the appearance of an Aurora Borealis-like light and flame issuing from the poles and sides of the magnet: this phenomenon is altogether invisible to ordinary eyes. In certain neuropathic diseases, especially catalepsy, a powerful attraction exists between the hand and the magnet; so that whichever way the latter is moved, the former follows, as if it were a bit of iron adhering to the instrument. When placed in the hand, the magnet was convulsively grasped, so that it was difficult to loosen the hold. Water, along which a magnet had been drawn, was found to possess the property of attracting the hand of a cataleptic patient. The same was true of other substances, when magnetised, (and even when unmagnetised, in many instances) besides water; all acted, more or less powerfully, upon her. It was afterwards discovered that none but *crystalline* bodies had the effect of causing the hand to close spasmodically upon them: amorphous substances were inert. The law is general that every crystal, natural or artificial, exerts a special action on the animal nerve; this action being greatest in neuropathic persons. The poles of the crystal emit a lambent light, visible only to sensitive persons; and the hand of such persons is distinctly attracted by it. Water also, and various other substances, may be magnetized (we use this word, for want of a better) with the point or pole of a crystal. In short the phenomena are altogether similar to those produced by the magnet. The magnetic needle, however, is not at all affected by any crystal. The *polar force*, therefore, which exists in *crystals*, and produces on healthy and diseased persons the above described effects, is not identical with the *magnetic force*. Both forces exist in magnets; in other words, a magnet is the seat of two forces, not one alone. The *crystalline force* passes through all solid bodies, but with different degrees of facility; it may also be transferred to other bodies, which may be charged with it by contact.

The same force, that is possessed by crystals and magnets, exists in the *human hand*. This, when passed over sensitive patients, acts upon them in the same manner; it exerts an attractive influence upon the hands of cataleptic patients; and it emits the same beautiful luminous phenomena—visible only to the sensitive—described above. In short the *crystalline force*, and (what has been called) *animal magnetism* " are absolutely one and the same; and therefore the laws, which regulate the former, admit of being fully applied to the latter."

Besides the three sources, hitherto mentioned, of this new power or force, the Baron has discovered that it resides in, or is generated by, other agencies and objects.—For example, the sun's rays, and the light of the moon* contain it in abundance; heat also, friction, and artificial light are, all, sources of it. One of the most prolific causes of the Magneto-Crystalline force is chemical action; and, as Digestion and Respiration are nothing but chemical processes, the Baron considers that these two functions are the great generators of it in the human body. The ghost-like luminous appearances observed by certain persons above graves, although unseen by most people that are in health, can now be readily understood: they are of a purely physico-chemical nature, being but one of the results of the

---

* " The action of the sun on sensitive patients agrees with that of magnets, of crystals, and of the human hand." * * * * * " Moonlight possesses a peculiar attraction, in virtue of which it acts on those affected with lunacy; nay, it may possibly cause that malady."

chemical action going on in the corpse.* "Many ghost-stories will now find their natural explanation."

As the Baron proceeded in his investigations, he found that all bodies whatsoever, in some degree, contain the new force, producing the sense of heat and cold to the sensitive, and emitting a luminous appearance in the dark,—the light being, however, variously coloured. It is, therefore, a universally diffused and active property of matter, although existing in variable and unequal distribution in different objects of the material world. Even the stars and planets are probably not without their influence, says our author, upon "our sublunary world, in practical matters, or in the working of the human brain."

Several other astounding facts (or fictions) remain to be noticed: what we have already specified will, however, suffice to give our readers a general idea of the (supposed) discovery of this new *force* in nature.

Dr. Gregory seems to be pretty confident of its truth. He tells us that, in the few instances in which he has repeated Reichenbach's experiments, he has found them entirely accurate; for example, that "crystals exert *an influence* on the human system in a large majority of persons; while in some, the sensitive, their action is exceedingly powerful." As a matter of course, much will be done in the course of the next three months, more especially by the believers in Animal Magnetism, in carrying out the subject; so that we shall probably have an opportunity of recurring to it in our next number. Meanwhile we hope that the result of their enquiries may not prove to be as fallacious as in the recent case of the Electrical Girl, that was at first vouched for by learned doctors and *savants* in the French Academy, and is now admitted to be all a hoax.

* Dr. Gregory remarks that we can now account for the "corpse-lights in church-yards, which were often visible to the gifted alone, to those who had the second sight, for example."

